# Scoping the Impact of Changes in Population Age-Structure on the Future Burden of Foodborne Disease in The Netherlands, 2020–2060

**DOI:** 10.3390/ijerph10072888

**Published:** 2013-07-11

**Authors:** Martijn Bouwknegt, Wilfrid van Pelt, Arie H. Havelaar

**Affiliations:** 1 Centre for Zoonoses and Environmental Microbiology, National Institute for Public Health and the Environment, P.O. Box 1, Bilthoven NL-3720BA, The Netherlands; E-Mail: arie.havelaar@rivm.nl; 2 Centre for Epidemiology and Surveillance of Infectious Diseases, National Institute for Public Health and the Environment, P.O. Box 1, Bilthoven NL-3720BA, The Netherlands; E-Mail: wilfrid.van.pelt@rivm.nl; 3 Institute for Risk Assessment Sciences, Faculty of Veterinary Medicine, Utrecht University, Utrecht NL-3508TD, The Netherlands

**Keywords:** incidence, mortality, future, ageing, The Netherlands, *Salmonella* spp., *Campylobacter* spp., *Listeria monocytogenes*, *Toxoplasma gondii*, hepatitis A virus

## Abstract

A demographic shift towards a larger proportion of elderly in the Dutch population in the coming decades might change foodborne disease incidence and mortality. In the current study we focused on the age-specific changes in the occurrence of foodborne pathogens by combining age-specific demographic forecasts for 10-year periods between 2020 and 2060 with current age-specific infection probabilities for *Campylobacter* spp., non-typhoidal *Salmonella*, hepatitis A virus, acquired *Toxoplasma gondii* and *Listeria monocytogenes*. Disease incidence rates for the former three pathogens were estimated to change marginally, because increases and decreases in specific age groups cancelled out over all ages. Estimated incidence of reported cases per 100,000 for 2060 mounted to 12 (*Salmonella*), 51 (*Campylobacter*), 1.1 (hepatitis A virus) and 2.1 (*Toxoplasma*). For *L. monocytogenes*, incidence increased by 45% from 0.41 per 100,000 in 2011 to 0.60 per 100,000. Estimated mortality rates increased two-fold for *Salmonella* and *Campylobacter* to 0.5 and 0.7 per 100,000, and increased by 25% for *Listeria* from 0.06 to 0.08. This straightforward scoping effort does not suggest major changes in incidence and mortality for these food borne pathogens based on changes in de population age-structure as independent factor. Other factors, such as changes in health care systems, social clustering and food processing and preparation, could not be included in the estimates.

## 1. Introduction

Demographic forecasts show that, as in many industrialized countries, the population in The Netherlands is aging [1]. This demographic shift may have consequences for morbidity and mortality caused by infectious diseases. Aging of humans is associated with, amongst others, changes to the innate and adaptive immune system, a process referred to as immunosenescence [2]. Although the number of immune cells does not decrease, functional alterations in some of the associated cell types occur [3]. These alterations likely cause changes in the defense mechanism to pathogens in the elderly, either due to an increased probability of infection and/or an increased probability to disease following infection. Another mechanism related to aging that could play a role in increased susceptibility of the elderly to infectious diseases is an alteration in the gastric hydrochloric acid secretion [4]. As gastric acid is the first in a series of defense mechanism against gastrointestinal pathogens, pH disruption likely leads to an increased probability of pathogen survival and transfer to the intestines. This effect on disease incidence is indirectly shown by the increased risk for gastrointestinal disease due to proton-pump-inhibitor use [5]. Looking at epidemiological data, specific foodborne infectious diseases are indeed observed more frequently in the elderly than in other age groups, such as *Listeria meningitis* [6]. Changes in the proportion of elderly might therefore result, amongst others, in an increase in listeriosis cases.

The effects of the demographic shift may also occur in the very young. A yet underdeveloped immune system, lack of acquired immunity or alternative behavior leading to different exposure to pathogens puts the very young at a higher risk for infection and/or disease. Pathogens such as *Salmonella* spp. and *Campylobacter* spp. indeed show the highest incidence rates among those aged 0–4 [7]. A decrease in the proportion of young individuals therefore might contribute substantially to a lower overall incidence for these bacteria.

A demographic shift towards a larger proportion of elderly people in the population and a smaller proportion of the young might thus have counteracting effects on infectious disease incidence at the population level. The aim of the current study was therefore to assess the effect of the demographic shift regarding age as independent factor on the incidence and excess mortality of foodborne diseases. The study focused on infectious diseases caused by five foodborne pathogens previously identified as causing either a high population and/or a high individual disease burden: *Campylobacter* spp., non-typhoidal *Salmonella* spp., *Listeria monocytogenes*, hepatitis A virus and acquired infections with *Toxoplasma gondii*. Perinatal *T. gondii* episodes were not considered.

## 2. Data and Methods

### 2.1. Population Data

Data on the current and expected future age-structure of the population for 2011 and per decade from 2020 to 2060 were obtained from Statistics Netherlands [1], based on the expected fertility of women, immigration rate, emigration rate, and anticipated birth and death rates. Data were grouped according to age: 0 years, 1–4, 5–9, (5-year classes), ≥90.

### 2.2. Incidence Estimation

The incidence and excess mortality are based on reported cases and not corrected for underreporting to population incidences. Future incidences of disease as function of the age-structure of the population were estimated from age-specific incidence rates per pathogen, assuming these rates remain constant until 2060. For *Campylobacter* spp. and *Salmonella* spp. these probabilities were calculated from the age of cases reported to RIVM through laboratory surveillance in 2011 [7].

Hepatitis A is a notifiable disease in The Netherlands and cases are reported to the Municipal Health Service and subsequently entered in a national database. The ages of reported cases were obtained from this database for the years 2007 through 2011. Ages of cases for *L. monocytogenes* were obtained through active surveillance in The Netherlands [8] from 2005 through 2011. As case numbers for hepatitis A and acquired listeriosis are relatively small, data for the study years were pooled per pathogen to more robustly estimate the disease risk for reported cases per age class.

For *Toxoplasma gondii* we used the seroconversion incidence approach as used by Havelaar *et al.* [9], who described the mean seroconversion rate to be 0.85% per year from approximately 20 years to 65 years of age based on data from Hofhuis *et al.* [10] for 2006 and 2007. The probability of infection in the other age classes was set to “0”.

The age-specific incidences were multiplied with the number of individuals per age class per study-year to obtain the expected number of cases for that age-class. These were subsequently summed over all age-classes for each study year and expressed as incidence per 100,000 population for comparison.

### 2.3. Excess Mortality Estimation

Excess mortality is defined in this study as the mortality that occurs additionally within 365 days after onset of the disease. Helms *et al.* [11] estimated the odds ratio for death due to laboratory confirmed salmonellosis and campylobacteriosis in regular surveillance to be 2.85 and 1.86, respectively. The approximate (age-independent) excess probability of death was calculated as (OR-1), amounting to 1.85 and 0.86, respectively. These numbers were multiplied with the average probability of death per 5-year age class according to Statistics Netherlands [1] and subsequently multiplied with the estimated number of reported cases per age class to obtain the estimated number of deaths due to foodborne infections [9].

Fatal listeriosis cases are reported to RIVM as part of the surveillance [8] and the age-specific risk of a fatal episode of listeriosis were obtained from these data. As the incidence of fatal acquired listeriosis is low, data from 2005–2011 were pooled to more robustly estimate the age-distribution of fatal cases. This age-distribution was subsequently applied to the average reported case numbers for 2005–2011 and divided by the population size per age-class for 2011 to obtain the estimated mortality rate per class.

Access mortality for *T. gondii* was not included in the current analysis, analogous to the approach of Havelaar *et al.* [9]. Ages for fatal HAV-cases were not available and could therefore not be considered.

## 3. Results and Discussion

### 3.1. Demographic Changes

**Figure 1:**
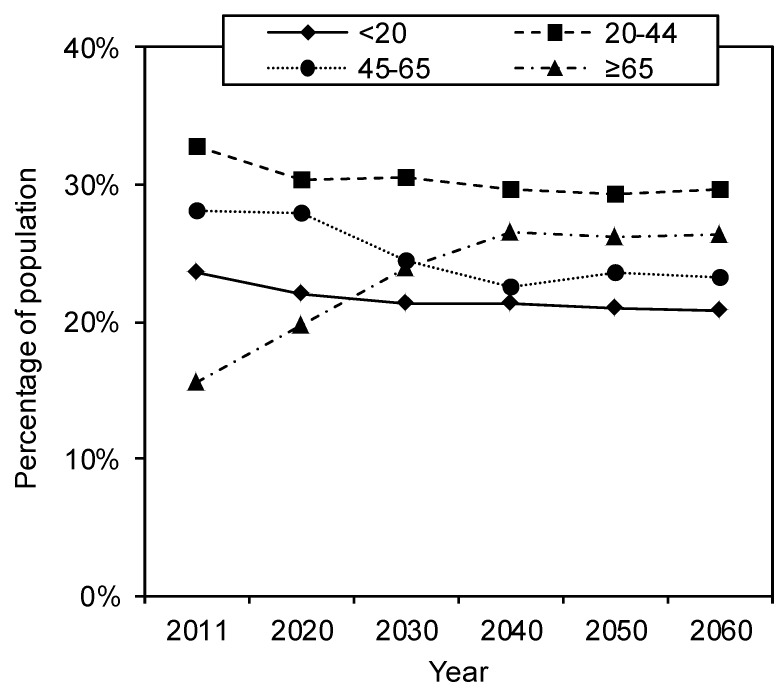
Demographic buildup in four age groups of the Dutch population between 2011 and 2060 according to counts (for 2011) and predictions (from 2020 onwards) by Statistics Netherlands [1].

The percentage of people ≥65 years increases from 16% in 2011 to 26% in 2040 and subsequently declines marginally to 25% in 2060 (Figure 1). The proportion of people aged 45 to 65 is expected to decrease more than those younger than 45. As the demographic shift is estimated to reach a new equilibrium around 2040, a particular emphasis in further descriptions of the results will be on 2040.

### 3.2. Incidence Estimation

**Figure 2:**
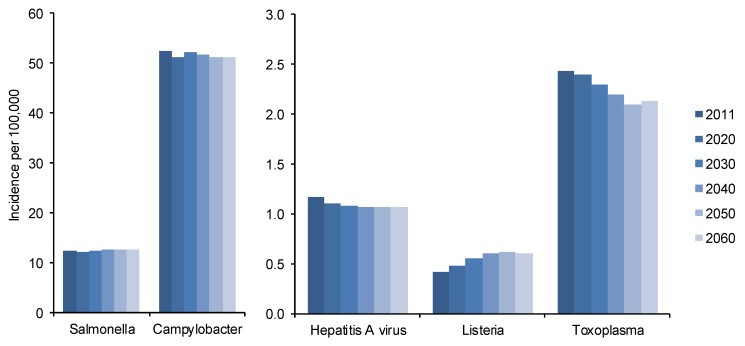
Estimated annual incidence per 100,000 population for five foodborne pathogens until 2060. The changes depicted are based on changes in population buildup regarding age as independent factor.

The age-specific probabilities of infection per year and pathogen are listed in Table 1. Relating these probabilities to the number of individuals per age class showed marginal overall changes in incidence for *Campylobacter* spp., *Salmonella* spp. and HAV (Figure 2). Larger changes per age-class were observed, but these cancelled out at population level. For *Salmonella* spp. and *Campylobacter* spp., for instance, the proportion of cases among those aged ≥65 increased by ~10% in 2040 but concurrently decreases with the same amount among the younger age classes. The largest decrease in the proportion was among those aged 0 to 20 for *Salmonella* spp. and those aged 45 to 65 for *Campylobacter* spp. (both −5%). The proportion of HAV cases among those aged ≥65 increased by 3%, and was associated with a decrease among the younger ages, especially those aged 45 to 65 (−2.2%).

**Table 1:** Estimated age-specific risks (%) for five important foodborne pathogens in The Netherlands.

Age	*Salmonella* ^1^	*Campylobacter* ^1^	HAV ^2^	*Listeria* ^3^	Fatal List ^3^	*Toxoplasma* ^4^
0	0.03	0.06	1.2 × 10^−4^	7.3 × 10^−5^	0	0
1–4	0.03	0.05	1.5 × 10^−3^	1.8 × 10^−5^	0	0
5–9	0.02	0.03	2.9 × 10^−3^	1.4 × 10^−5^	0	0
10–14	0.01	0.03	2.2 × 10^−3^	0	0	0
15–19	0.02	0.07	1.6 × 10^−3^	6.7 × 10^−5^	0	0
20–24	0.02	0.10	1.8 × 10^−3^	9.1 × 10^−5^	0	0.85
25–29	0.01	0.06	1.3 × 10^−3^	1.8 × 10^−4^	0	0.85
30–34	0.01	0.04	1.0 × 10^−3^	2.3 × 10^−4^	0	0.85
35–39	0.01	0.04	1.6 × 10^−3^	2.5 × 10^−4^	0	0.85
40–44	0.01	0.04	1.2 × 10^−3^	5.2 × 10^−5^	1.1 × 10^−5^	0.85
45–49	0.01	0.04	9.4 × 10^−4^	1.5 × 10^−4^	1.1 × 10^−5^	0.85
50–54	0.01	0.06	7.8 × 10^−4^	2.3 × 10^−4^	8.4 × 10^−5^	0.85
55–59	0.01	0.06	7.0 × 10^−4^	4.5 × 10^−4^	1.2 × 10^−4^	0.85
60–64	0.01	0.05	4.1 × 10^−4^	5.8 × 10^−4^	1.8 × 10^−4^	0.85
65–69	0.01	0.06	2.8 × 10^−4^	1.0 × 10^−3^	1.6 × 10^−4^	0
70–74	0.01	0.06	1.4 × 10^−4^	1.6 × 10^−3^	3.1 × 10^−4^	0
75–79	0.02	0.06	2.6 × 10^−4^	1.9 × 10^−3^	3.1 × 10^−4^	0
80–84	0.02	0.04	3.0 × 10^−4^	2.7 × 10^−3^	1.6 × 10^−4^	0
85–89	0.01	0.05	1.0 × 10^−4^	2.0 × 10^−3^	0	0
≥90	0.01	0.02	6.9 × 10^−4^	1.9 × 10^−3^	1.5 × 10^−4^	0

^1^ based on cases reported in laboratory surveillance in 2011; ^2^ based on cases reported in passive surveillance between 2007–2011 (pooled); ^3^ based on cases cases reported in active surveillance between 2007–2011 (pooled); ^4^ based on seroconversion as detailed in Havelaar *et al.* [9].

The estimated incidence of listeriosis increased by 45% from 0.41 per 100,000 in 2011 to 0.60 per 100,000 in 2040. The proportion of cases increased among those aged ≥65 by 15%, and was countered predominantly by a decrease among those aged 45 to 65 (−10%). The estimated incidence of toxoplasmosis increased among those aged 20 to 29 and 60 to 65 by 7% and 20% respectively, but decreased among those aged 30 to 65 and lead to the overall decrease of 10% by 2040.

These results indicate that the change in age-structure of the population, as independent factor, does not lead to large changes in the overall incidence. The shifts in age-structure were predicted to cause an increase in some age-groups (older) while a decrease was predicted for other age-groups. Over all ages, these differences cancel each other out. This approach was however intended as scoping effort, with focus on a change in a single factor (*i.e.*, the age-structure of the population). Aspects that were not considered in this analysis are for instance a change in the age-specific probabilities of disease. A change in health care availability, accessibility and performance might change these probabilities considerably. Prescribing proton-pump inhibitors, as is done particularly to the elderly, is likely related to greater susceptibility to gastrointestinal disease [5,12]. Changes in the use of such medication in future years can change the age-specific probability of infection compared to the current baseline for 2011.

Another aspect that is not considered in the current study is a dynamic change in immunity. Infection risks for HAV, for instance, have declined steadily in the past decades due to increased vaccine-induced protection and increased socio-economic and sanitation conditions [13,14]. McDonald *et al.* [15] predicted that the HAV seroprevalence declines in The Netherlands by 2030, leaving a larger proportion of the population susceptible to infection. In that study, scenario-based predictions on disease burden for HAV showed a three-fold increase in 2030 when assuming a constant infection probability, as was done in the current study, whereas a further decreasing infection risk at the rate observed in past decades was estimated to decrease the HAV disease burden five-fold [15]. The difference between results of that first scenario and our results, both considering a constant incidence, might be caused by the different measure estimated. Where our estimates considered incidence, McDonald *et al.* [15] additionally considered the severity of infections by estimating disability adjusted life years. An estimated increase in incidence among the elderly combined with an increased risk for severe complications due to hepatitis A in the elderly compared to younger age classes caused the DALY estimate to increase.

Like for HAV, a decreasing trend in prevalence of *Toxoplasma gondii* IgG is observed in the Netherlands from 46% in 1987 to 35% in 1995/1996 and 19% in 2007/2008 among women of reproductive age [10]. This decrease may lead to increased susceptibility to infection when exposed in the coming decades. The exposure may however also decrease. The observed seroprevalence decrease in the past 20 years has been attributed to changes in farming systems and increased consumption of frozen meat by consumers [16]. We can therefore not assess whether our assumption of constant infection probability lead to an over- or an underestimation of the future disease risks due to *Toxoplasma gondii*.

Also, the implementation of effective intervention measures to reduce exposure of the population to a pathogen may decrease the age-specific probabilities. Depending on the targeted population these differences can affect particular age groups or the entire population. The current study thus assumes a simplistic situation in which all aspects of society remain unchanged except for the age-related structure of the population. The current incidence estimates therefore do not bear predictive values, but are useful in understanding the expected effect of population ageing as independent factor on foodborne pathogens.

### 3.3. Excess Mortality Estimation

**Figure 3:**
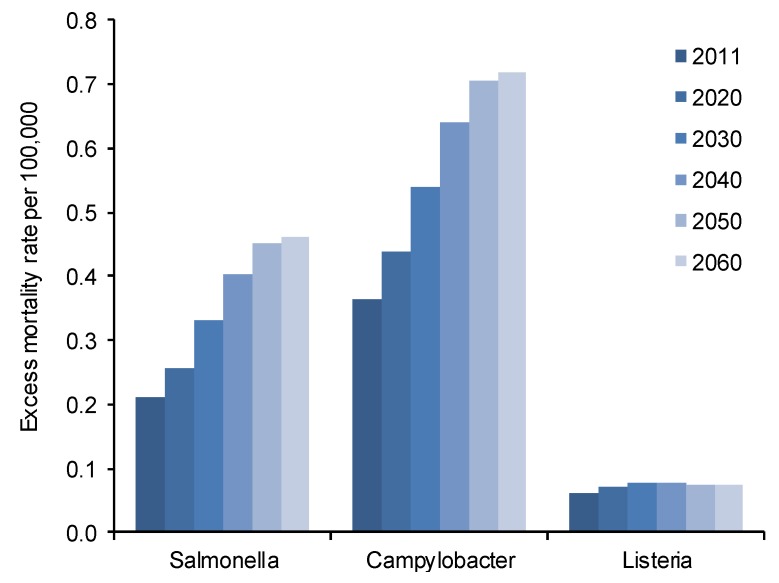
Estimated excess mortality over all ages per 100,000 population due to disease from three foodborne pathogens until 2060. The changes depicted are based on changes in population buildup regarding age as independent factor.

Excess mortality peaked in 2060 and the rate approximately doubled from 0.21 to 0.46 per 100,000 population for *Salmonella* spp. and from 0.36 to 0.7 per 100,000 population for *Campylobacter* spp. (Figure 3). The excess mortality was observed among the people aged ≥65 and remained approximately constant in younger age groups for both pathogens.

The estimated excess mortality rate due to *L. monocytogenes* increased by 25% from 0.061 to 0.078 per 100,000 population. This increase was found among those aged 65 and over. The rate among younger individuals was estimated to remain approximately unchanged.

Similar to the future incidence estimation, the excess mortality estimates consider current conditions to remain unchanged until 2060. A change in health care availability, accessibility and performance might change the excess probabilities of death. Improved treatment of severe cases can increase the likelihood for recovery and hence lower the excess probability of death. Such future changes are obviously not available for inclusion in such estimation, but need to be considered when interpreting the results.

## 4. Conclusions

The average age of the Dutch population is predicted to increase until 2060, with a peak of the proportion elderly aged ≥65 in 2040. Estimated disease incidence rates for *Campylobacter* spp., *Salmonella* spp., hepatitis A virus and *Toxoplasma gondii* nevertheless will change marginally until 2060, because increases and decreases in specific age groups cancel out over all ages. For *L. monocytogenes*, incidence estimates will increase from 0.4 per 100,000 in 2011 to 0.6 per 100,000 in 2040. Estimated mortality rates over all ages will increase two-fold for *Salmonella* and *Campylobacter*, and increase by 25% for *Listeria*. This straightforward scoping effort does not suggest major changes in incidence of foodborne pathogens based on demographic changes in age-structure as independent factor. Other factors, such as changing policies, changes in social clustering of the elderly or changing healthcare systems have not been considered in these estimates and may be more influential. However, prognoses for such factors are not currently available.
